# A New Paradigm for Understanding and Enhancing the Critical Heat Flux (CHF) Limit

**DOI:** 10.1038/s41598-017-05036-2

**Published:** 2017-07-12

**Authors:** Abdolreza Fazeli, Saeed Moghaddam

**Affiliations:** Department of Mechanical and Aerospace Engineering, University of Florida, Gainesville, FL 32603 USA

## Abstract

Nearly a century of research on enhancing critical heat flux (CHF) has focused on altering the boiling surface properties such as its nucleation site density, wettability, wickability and heat transfer area. But, a mechanism to manipulate dynamics of the vapor and liquid interactions above the boiling surface as a means of enhancing CHF has not been proposed. Here, a new approach is implemented to limit the vapor phase lateral expansion over the heat transfer surface and actively control the surface wetted area fraction, known to decline monotonically with increasing heat flux. This new degree of freedom has enabled reaching unprecedented CHF levels and revealed new details about the physics of CHF. The impact of wickability, effective heat transfer area, and liquid pressure on CHF is precisely quantified. Test results show that, when rewetting is facilitated, the CHF increases linearly with the effective surface heat transfer area. A maximum CHF of 1.8 kW/cm^2^ was achieved on a copper structure with the highest surface area among all tested surfaces. A model developed based on the experimental data suggests that the thermal conductivity of the surface structures ultimately limits the CHF; and a maximum CHF of 7–8 kW/cm^2^ may be achieved using diamond surface structures.

## Introduction

Boiling is a ubiquitous mechanism of heat transfer with numerous applications ranging from small-scale HVAC and refrigeration systems used in most buildings to large boilers in energy and process industries. Due to its unique performance characteristics, boiling has been implemented in extremely demanding applications such as fusion reactors^1–3^. The ebullition process in boiling triggers a set of heat and mass transfer events that can generate extremely high local cooling rates^4–6^. As such, in response to demands for removing heat from confined spaces in modem applications^7–10^, the boiling science community has embarked on extensive studies^11–13^ to implement boiling in heat exchangers with an order of magnitude smaller footprint than their traditional counterparts. What has made this task challenging is the unusual heat flux levels encountered in some of the advanced electronics (high performance CPUs, X-band radars, power electronics, etc.). For example, high voltage MOSFET and diode chips^14–16^ and GaN MMICs (Monolithic Microwave ICs)^17, 18^ can generate waste heat rates as high as 1000 s W/cm^2^.

To address this challenge, numerous studies have focused on enhancing the boiling critical heat flux (CHF) limit^19–33^. CHF, a century-old known limit associated with the boiling heat transfer process, is the highest heat flux a surface can exchange with a boiling fluid before a vapor layer isolates the surface from the liquid (i.e. prevents surface rewetting). Since the inception of the boiling science through introduction of the boiling curve and CHF by Nukiyama^34^, numerous pioneering scientists^35–39^ have attempted to understand the physical nature of CHF and enhance its limit. A variety of enhancement techniques have been attempted and different hypotheses have been presented to explain the experimental findings. CHF enhancement has been attributed to different parameters such as increased nucleation site density^22, 29^, contact line length^24, 26^ and ability of the surface to rewet via capillary wicking^19, 27–30^.

The most recent attempts on CHF enhancement involve implementation of surface micro- and nanostructures. Studies are consistently showing an increase in CHF beyond predictions of the existing CHF models suggesting new physics and possibilities for substantial increase in CHF. Notably, Wei and Chen^31^ investigated the effect of micropillars on pool boiling of FC-72 and observed substantial improvement in CHF. Chen *et al*.^22^ studied pool boiling of water on silicon and copper nanowires and observed CHF values as high as 200 W/cm^2^. More recently, Chu *et al*.^24, 26^ reported a maximum CHF value of 250 W/cm^2^ on hierarchical structures. One of the main challenges in deciphering the physics of CHF on micro- and nanostructures is that these structures impact nearly all factors that are suggested to affect CHF such as surface wettability, wickability, contact line length and effective heat transfer area. The difficulty lies in isolating each one of these parameters to find their contribution to CHF enhancement. The chaotic nature of the flow field and rapid spatial and temporal variations of thermal events at the solid-fluid interface greatly limit the ability to conduct measurements necessary for explaining the CHF sub-processes.

In the absence of adequate microscale measurement techniques, the existing studies have attempted to correlate the CHF limit of a surface to its structural and physical properties. For example, Chu *et al*.^24, 26^ suggested that roughness-amplified capillary forces are responsible for the CHF enhancement on structured surfaces. In a recent comprehensive study, Rahman *et al*.^25^ carefully engineered nearly forty surfaces with different wickability levels and clearly demonstrated that CHF increases linearly with the surface wickability. They reported a maximum CHF of 260 W/cm^2^ on a hierarchical structure with the highest wickability. Shortly after Rahman *et al*.’s study^25^, Dhillon *et al*.^20^ showed that CHF increases with the surface structures wickability, albeit not linearly, and reported a maximum CHF of ~200 W/cm^2^ on a microstructured surface. It should be noted that in both studies, the *wicking rate* of the fluid was not directly measured during the boiling process. Rahman *et al*.^25^ measured the wickability statically in an *ex-situ* experiment and Dhillon *et al*.^20^ estimated the wickability using a theoretical liquid wicking model. However, on a boiling surface, bubbles emerge from surface structures and interrupt the wicking process (e.g. by pushing against the wicking liquid).

In this paper, a new approach is introduced to control the CHF dynamics and reach unprecedented CHF levels. In contrast to the existing studies that are focused on better engineering of the heated surface, the main innovation here involves installation of a superhydrophobic vapor permeable wall (i.e. a membrane) several hundred microns away from the heated surface. As described in details in the next section, when a bubble comes in contact with the hydrophobic surface, a contact region forms between the two and rapidly expands. Forces generated as a result of this phenomenon pull the bubble away from the heated surface. This new degree of freedom in manipulating hydrodynamics of the vapor and liquid flow above the heated surface allows controlling the surface wetted area fraction that has been observed to decline monotonically with increasing heat flux^40–43^. In the following sections, first, fundamentals of the new concept are introduced through an adiabatic visualization study that illustrates the utilization of surface tension and pressure forces to remove bubbles from immediately above the nucleation site, such that the surface is rapidly rewetted. Then, the architecture of a set of heat sinks with different surface structures is introduced and their CHF performance is analyzed. Lastly, a fully deterministic model is introduced that can accurately predict the experimental results. This model is then used to predict the highest achievable CHF.

## Proposed Concept

**Figure 1:**
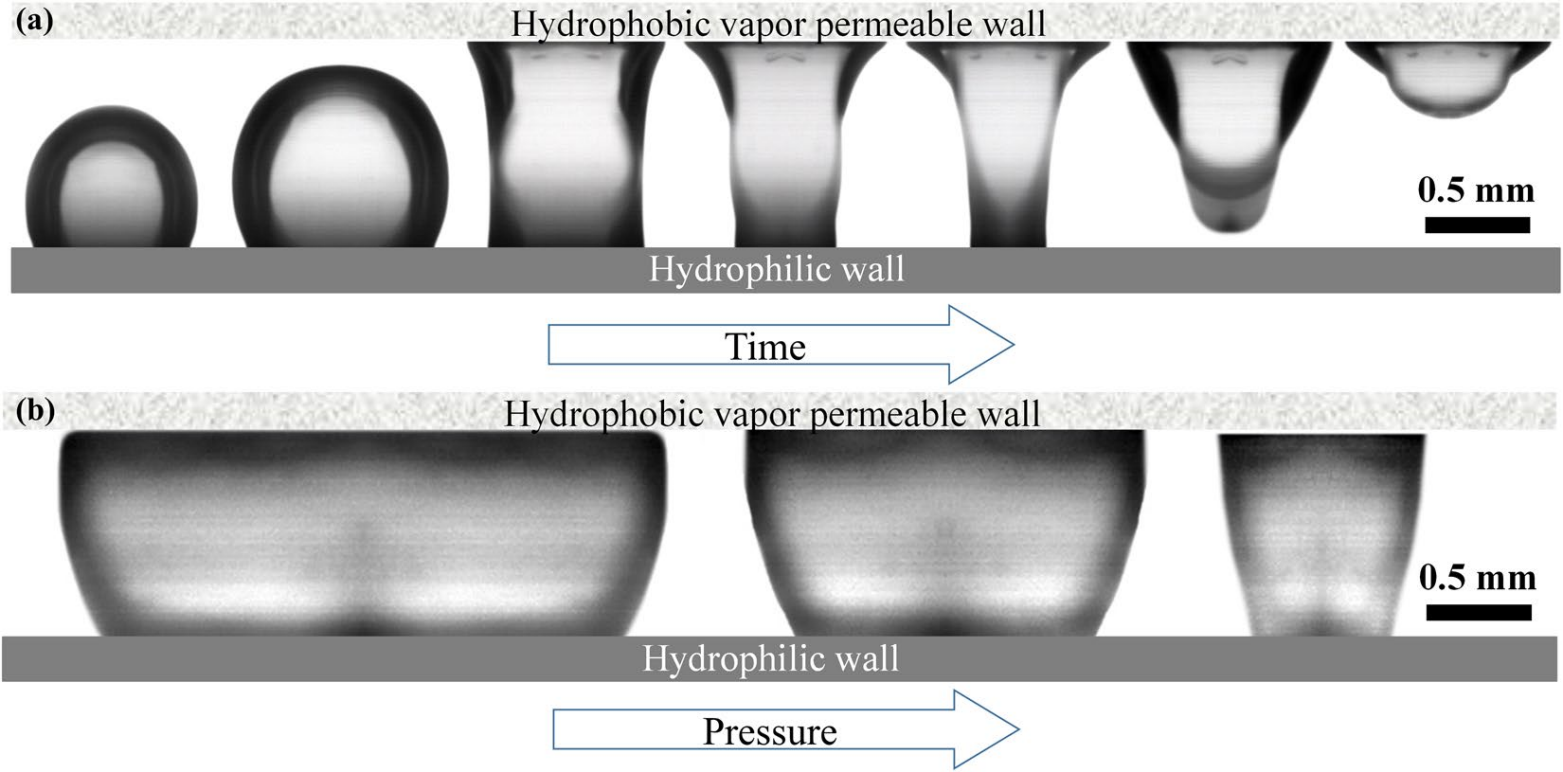
Bubble extraction from a channel filled with water as a function of (**a**) time and (**b**) channel pressure.

To demonstrate the working principle of the method, a set of adiabatic visualization tests are conducted. Figure 1 shows the side view of a channel formed between a hydrophilic silicon wall (bottom) and a hydrophobic nanofibrous PTFE wall (top). The sidewalls are made from optically clear polycarbonate. The hydrophobic wall allows gas (or vapor) to exit the channel while constraining the liquid. The channel is filled with water and pressurized. First, air is injected into the channel using a syringe pump at a constant rate (100 ml/hr), via a 5 μm through-hole made within the silicon wall (to resemble nucleation from a cavity). As seen in Fig. 1a, as soon as the bubble comes in contact with the hydrophobic surface, a contact region forms between the two and expands. The forces generated as a result of this phenomenon pull the bubble away from the hydrophilic surface. Subsequently, the contact line recedes over the hydrophilic surface until the bubble snaps off, and liquid fully rewets the hydrophilic surface.

In a subsequent test, designed to resemble a significant vapor generation rate and surface vapor coverage, the size of the through-hole was increased to greatly enhance the air injection rate such that the bubble could laterally expand before exiting through the vapor permeable wall. Tests conducted at three different pressures show (Fig. 1b) that bubble coverage of the surface is reduced (i.e. the wetted area is increased) as the channel pressure is increased. In this condition, the liquid pressure acting on the outer surface of the bubble prevents it from expanding laterally and forces the vapor to exit through the membrane. While surface tension controls dynamics of the bubble deformation and contact line movements over the surfaces, the pressure potential controls the rate of bubble exit through the vapor permeable wall.

**Figure 2:**
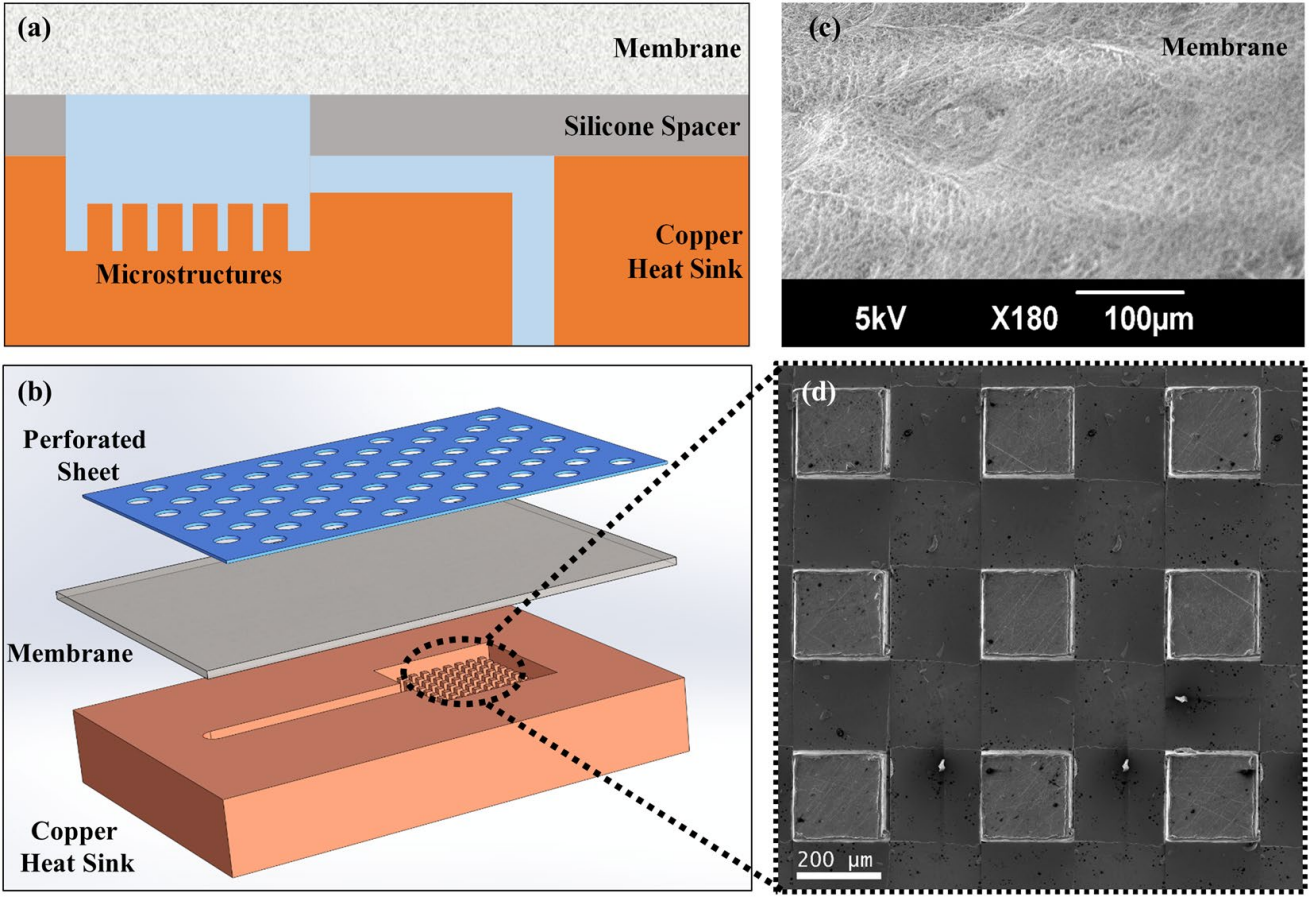
Schematic of test device showing (**a**) cross sectional view of copper base plate, silicone spacer and hydrophobic PTFE membrane and (**b**) isometric view of the device assembly; and SEM micrograph of (**c**) PTFE membrane and (**d**) a surface structure.

The physics of the process discussed above can be exploited to enhance the CHF in two-phase microchannel heat sinks. The key towards achieving this goal is to design a heat sink that enables subjecting the bubble-liquid interface to an omnidirectional pressure such that the bubble growth over the heat transfer surface becomes limited. This requires, unlike conventional two-phase heat sinks discussed in the literature, to have no liquid exit. Figure 2a,b show a heat sink architecture that implements the introduced concept. Unlike other two-phase heat sinks that have a liquid inlet and a two-phase flow outlet, this heat sink has only one liquid connection (i.e. an inlet). The connection supplies the liquid to the active area of the heat sink populated by surface structures (Fig. 2b,d). A hydrophobic membrane (Fig. 2c) installed on the entire device confines the liquid pool while allowing the vapor to pass through. This arrangement of the liquid and vapor flow (i.e. separation of the liquid and vapor paths) and a vapor exit quality of 100% independent of the heat load constitute a fundamental departure from the existing heat sink architectures.

## Experimental Studies

The preliminary experiments were all conducted on copper heat sinks fabricated using a CNC machine. The copper heat sinks were then brazed to a heating block that provides the heat input and enables measurement of the heat flux and surface temperature. Water was used as the working fluid and was pumped into the device using a piezoelectric micropump (Model MP6, manufactured by Bartels Mikrotechnik GmbH) (see section S1 in the Supplementary section for more details on the experimental setup). To maximize the surface wettability, two approaches were first examined including chemical etching of copper nanowires^44^ and thermal growth of a rough oxide layer^45^. Both approaches produced nearly the same results, reducing the water contact angle to less than 5 degrees.

Three sets of experiments were designed to study the effect of membrane properties, surface structures wickability and heat transfer area on CHF. The studies on the effect of membrane properties were conducted using three different membranes. To study the effect of surface wickability and heat transfer area on CHF, five heat sinks were designed and fabricated. Table 1 provides surface structures dimensions for each one of the heat sinks as well as a metric of their wickability and surface area enhancement ratio. Surface structure wickability is considered as the ability of a structure to wick liquid over a distance because of capillarity. Measurement of the actual mass flux within surface structures on a boiling surface is an extremely difficult task and a unique parameter as a measure of wickability has yet to be defined. In the absence of a clear definition, two different approaches have recently been utilized. Rahman *et al*.^25^ quantified wickability of their surface structures using a method^46^ in which the surface structure is slowly raised to contact a pendant water droplet attached to a small-diameter capillary tube. The wickability was then defined as the volume flux of the liquid into the structure (a phenomenological parameter representing the effect of all parameters impacting wickability). In another approach, Dhillon *et al*.^20^ utilized an technique commonly used in the heat pipe wick literature that involves estimating the structure capillary pressure (*P*_*c*_) and permeability (*K*_*wick*_) using existing models. The liquid flux is then calculated using Darcy’s law $({\dot{m}}_{wick}^{^{\prime\prime} }=\rho {K}_{wick}{P}_{c}/\mu L)$, where *ρ* and *μ* denote liquid density and viscosity, respectively. One issue with this approach is that a wicking length is required to calculate $\dot{m}{^{\prime\prime} }_{wick}$. Dhillon *et al*.^20^ used an *ad hoc* wicking length of 2 mm at CHF. In this study, the ability of a surface to wick a liquid is simply defined as the product of its permeability and capillarity (*K*_*wick*_ *P*_*c*_) to avoid any assumption. This value for different surface structures is calculated using two models that were experimentally verified in prior studies^47–50^ (see section S2 in the Supplementary section for more details).

**Table 1:** Physical properties and wickability of surface structures.

Design	Spacing (S) [μm]	Width (W) [μm]	Height (H)[μm]	*K*_*wick*_ *P*_*C*_ × 10^6^ (Pa.m^−2^)	Enhanced Area (A_r_)
1	75	50	50	**0.151**	1.64
2	200	100	150	**0.453**	1.67
3	300	50	325	**0.779**	**1.56**
4	300	350	350	0.754	**2.16**
5	200	150	500	0.757	**3.45**

Another property of a surface structure that is hypothesized to affect CHF is its enhanced area ratio (*A*_*r*_) defined as the total area of the structure divided by its projected area. For surface structures made of square pillars with a width of *W*, a height of *H* and a spacing of *S*, it can be shown that *A*_*r*_ = 1 + 4*WH*/(*S* + *W*)^2^.

As can be seen in Table 1, structures #1 to 3 are designed with different *K*_*wick*_ *P*_*c*_ and a nearly constant *A*_*r*_ while structures #3 to 5 have a similar *K*_*wick*_ *P*_*c*_ but different *A*_*r*_.

### Effect of Membrane Permeability

**Figure 3:**
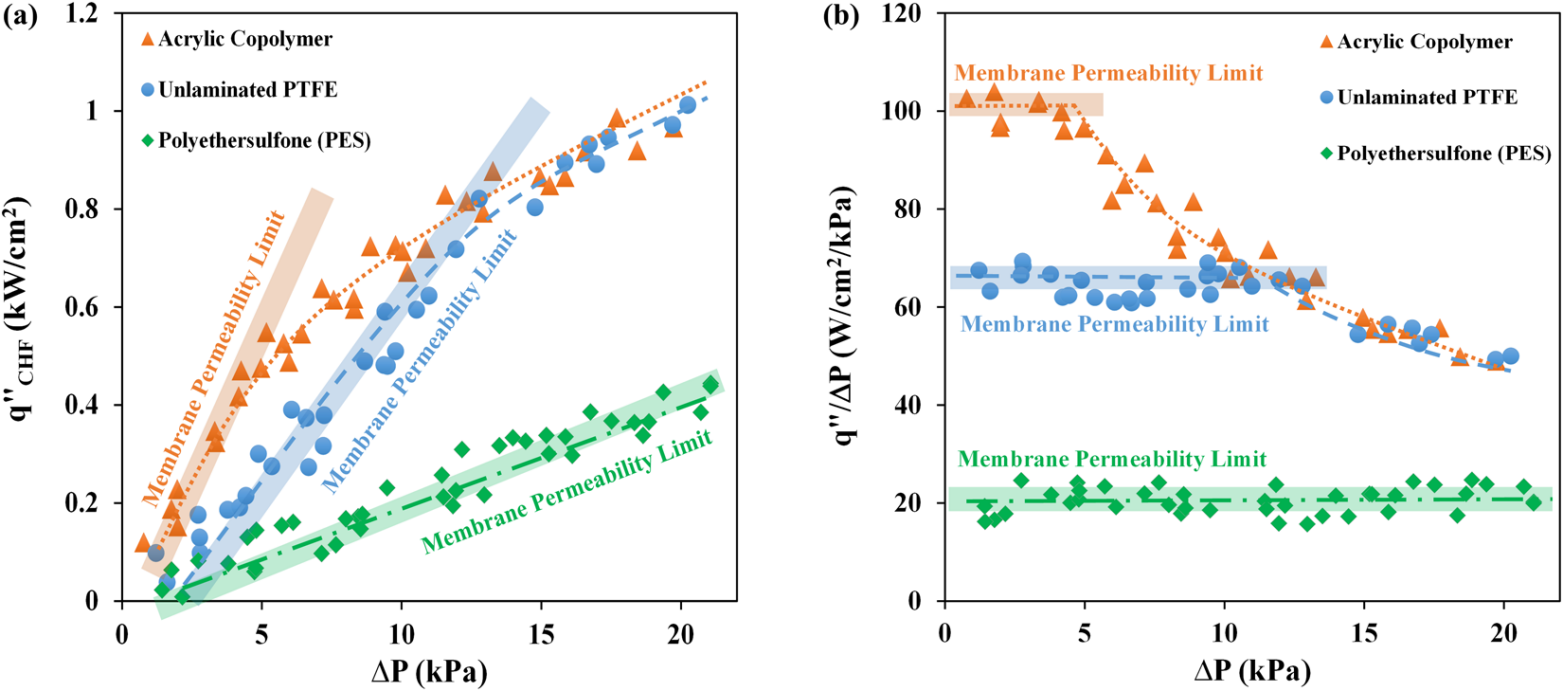
CHF performance of structure #4 tested with three different membranes; (**a**) Effect of membrane permeability on CHF and (**b**) CHF divided by liquid pool pressure as a function of liquid pressure.

The permeability and differential pressure across the membrane determine the rate of gas/vapor transport through the membrane^51–53^. To demonstrate the impact of membrane permeability on CHF, test results on a heat sink (design #4) with three different membranes are provided in Fig. 3a (see Table s-1 in the Supplementary section for more details on membrane properties). The graph shows CHF values at different applied pressures (gauge pressure, with respect to the vapor space, measured at the heat sink inlet). The data suggest that the membrane permeability has a significant impact on CHF. With high permeability membranes (acrylic copolymer and PTFE), this effect is limited to low pressures and performance values converge at high liquid pressures, where the applied pressure is high enough to push the generated vapor through the membrane. However, membrane permeability limits the CHF over the entire test conditions, when membrane permeability is the lowest (with PES membrane).

This phenomenon can be explained by comparing the vapor generation rate at the CHF, ${\dot{{\rm{m}}}}_{{\rm{CHF}}}^{^{\prime\prime} }(={{\rm{q}}}_{{\rm{CHF}}}^{^{\prime\prime} }/{{\rm{h}}}_{{\rm{fg}}}$) with the membrane mass transfer limit ${\dot{{\rm{m}}}}_{{\rm{mem}}}^{^{\prime\prime} }(={{\rm{\rho }}}_{{\rm{v}}}{{\rm{K}}}_{{\rm{mem}}}{\rm{\Delta }}{\rm{P}}$, where K_mem_ and ρ_v_ are the membrane permeability and vapor density at saturation pressure, respectively). While ${\dot{{\rm{m}}}}_{{\rm{CHF}}}^{^{\prime\prime} }$ depends on surface structures and applied pressure, ${\dot{{\rm{m}}}}_{{\rm{mem}}}^{^{\prime\prime} }$ is a function of membrane properties (i.e. pore size and thickness) and ΔP. If ${\dot{{m}}}_{{mem}}^{^{\prime\prime} }$ is higher than ${\dot{{m}}}_{{CHF}}^{^{\prime\prime} }$, CHF will not be limited by the membrane transport limit but by the surface structure itself; however, at low ${\dot{{m}}}_{{mem}}^{^{\prime\prime} }$ (which is the case for membranes with low permeability values) the CHF will be governed by membrane properties and not the geometry of microstructures. Figure 3b shows another way of illustrating this principle. In the pressure range that CHF is limited by the membrane permeability (i.e. the highlighted range), the ratio of CHF to applied liquid pressure (${q}_{CHF}^{^{\prime\prime} }/\Delta P$) is almost constant.

**Figure 4:**
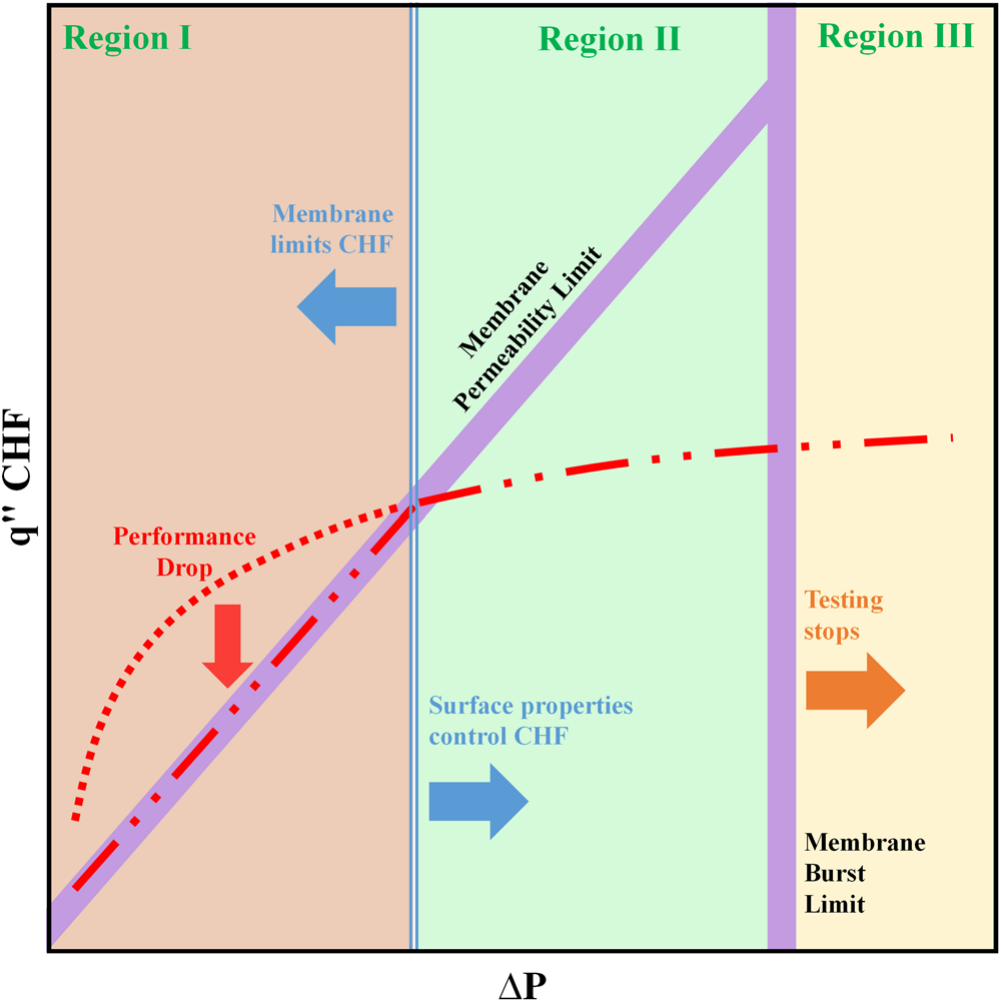
A schematic graph showing the main factors limiting the CHF depending on pool pressure (membrane is the limiting factor at Regions I and III).

Another membrane property that impacts the operational range of the heat sink is the membrane breakthrough pressure. The breakthrough pressure is a pressure at which the liquid is no longer constrained within the liquid pool and enters the membrane pores. Considering different factors, Fig. 4 is prepared to show regions at which each one of the introduced factors limits the CHF. At Region I, membrane permeability limits the maximum amount of heat that can be dissipated from the surface (${\dot{{\rm{m}}}}_{{\rm{mem}}}^{^{\prime\prime} }$ < ${\dot{{\rm{m}}}}_{{\rm{CHF}}}^{^{\prime\prime} }$). Improving membrane permeability is then considered an effective approach to enhance the CHF in this region. Region III represents the test conditions at which the liquid pressure exceeds the burst pressure of the membrane. Here, the liquid leaks from the membrane and experimental data can not be recorded. The only scenario under which the CHF limit is determined by the surface structures is illustrated in Region II. In this region, the maximum permissible flow of vapor through the membrane is more than the amount of vapor generated on the surface (${\dot{{\rm{m}}}}_{{\rm{mem}}}^{^{\prime\prime} }$ < ${\dot{{\rm{m}}}}_{{\rm{CHF}}}^{^{\prime\prime} }$) and CHF can be altered by adjusting the structure design.

### Effect of Structure Wickability

**Figure 5:**
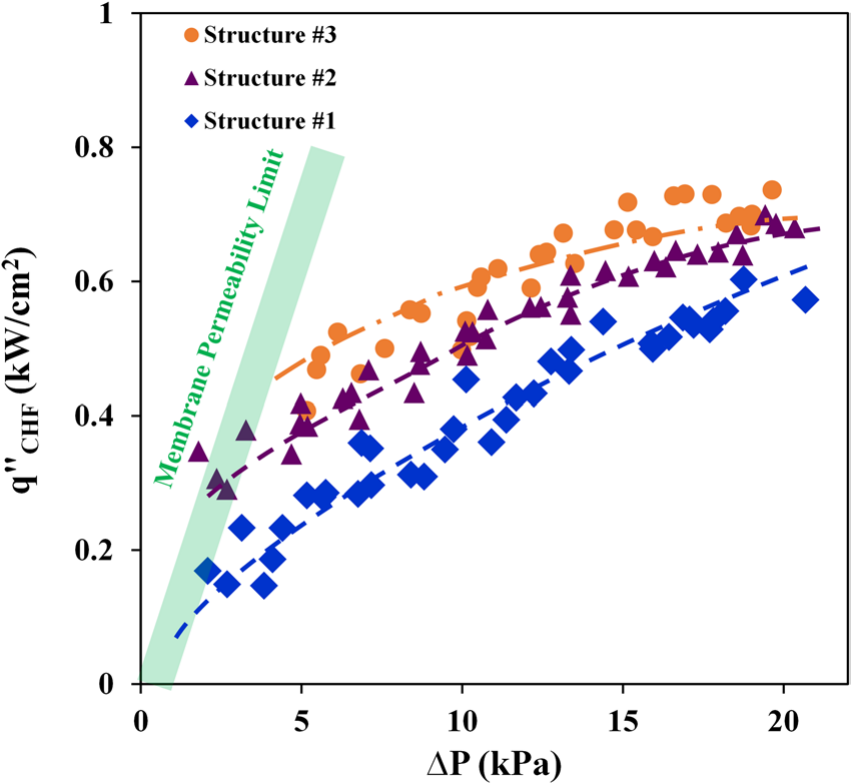
CHF as a function of applied liquid pressure.

In this section, the effect of surface wickability on CHF is analyzed by comparing the performance of structures #1 to 3. As mentioned earlier, these structures have different ${K}_{wick}{P}_{c}$ and a similar *A*_*r*_. As the results in Fig. 5 suggest, the CHF performance of these structures at low pressure is substantially different, clearly showing the impact of wickability on CHF as suggested in recent studies^20, 25^. The test results suggest that the CHF performance of all structures improves with increasing pressure. This trend is believed to be due to a decrease in the wicking length, since bubbles and vapor columns are squeezed, as illustrated in Fig. 1. The experimental data also show that the performance of structure #3 reached a plateau at high pressures (changes in CHF are statistically insignificant over the 16–20 kPa pressure range). Performance of structure #2 also reached a plateau but closer to 20 kPa. It is believed that the performance of structure #1 would also reach a plateau at a slightly higher pressure but the breakthrough pressure of the existing membrane prevented testing at a higher pressure.

The fact that increasing the pressure beyond a certain value does not enhance CHF (i.e. does not facilitate surface rewetting) may be explained by revisiting the concept of wickability on a boiling surface. As mentioned in the introduction section, the recent studies that have related the surface wickability to its CHF have measured or modeled liquid wickability of the structure in the absence of the ebullition process. However, on a boiling surface, bubbles formation and growth interfere with the liquid wicking process. As the nucleation frequency and bubbles growth rate increase with increasing CHF, the time period during which the liquid can rewet the surface declines monotonically. Therefore, if the bubble growth timescale becomes shorter than the rewetting timescale, the liquid can no longer rewet the surface. In our experiments, this limit may have been reached at heat flux values of 600–700 W/cm^2^ that are approximately 3 times the highest CHF values reported in prior pool boiling studies^20, 24–26^.

Additionally, Fig. 5 shows that at the highest pressure, maximum CHF values for structures #2 and #3 are less than 10% different. The CHF of structure #1 is also within 15% of the maximum (expected to rise at higher pressures). This is quite fascinating given the substantial difference in every dimension of these structures. It should be reminded that the wickability of these structures is significantly different and the only geometrical parameter that is the same for these structures is *A*_*r*_. In the following section, it is shown that increasing *A*_*r*_ linearly increases the CHF until another limit, which is the thermal conductivity of the structures material, is reached.

### Effect of Heat Transfer Surface Area

**Figure 6:**
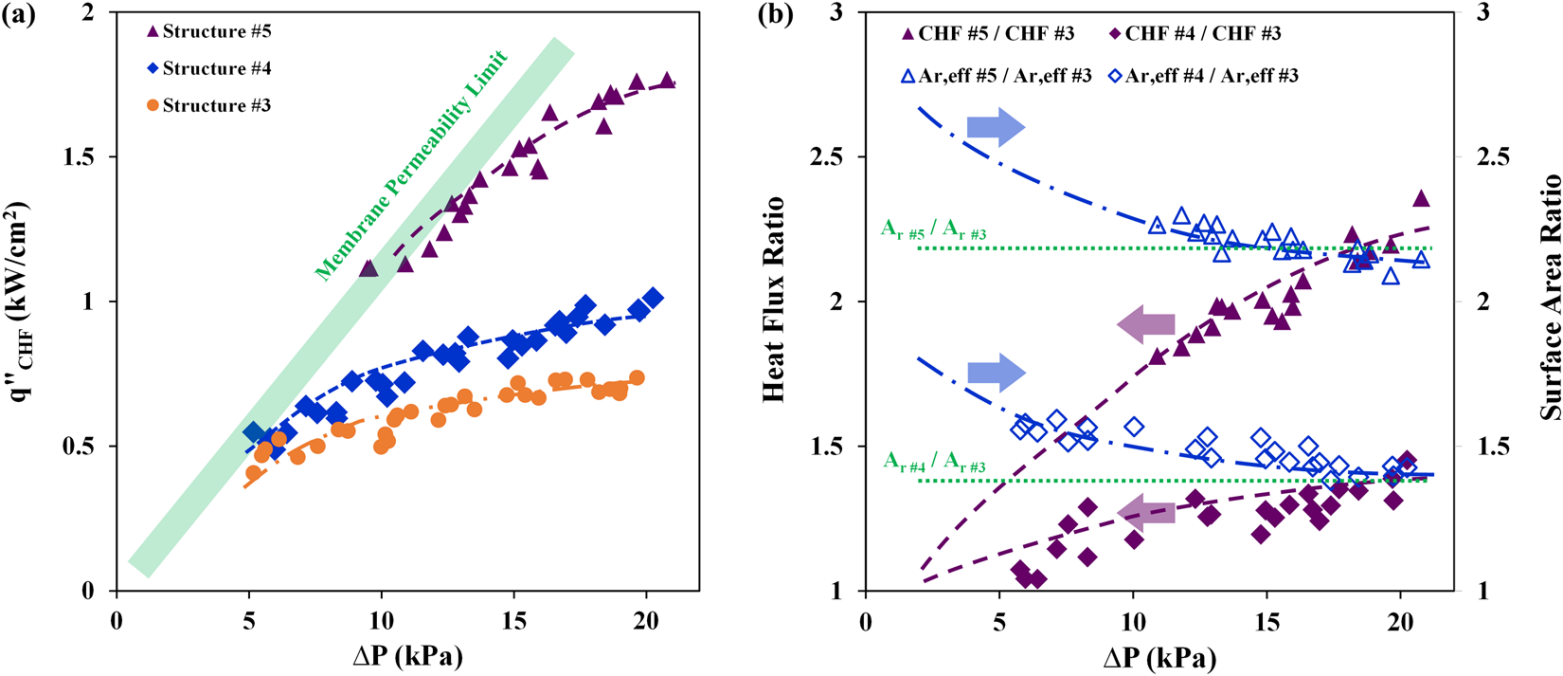
Effect of enhanced heat transfer area; (**a**) CHF as a function of applied liquid pressure and (**b**) CHF ratios of different structures as a function of pressure and comparison with enhanced area ratios of the corresponding structures (the experimental values are linearly extrapolated to estimate values at lower pressures).

A comparison of the test results on structures #3 to 5 are provided in Fig. 6. While the static wickability of these structures is identical, their *A*_*r*_ varies from 1.56 to 3.54. Similar to the trend seen on other surface structures (Fig. 5), the CHF values enhance with increasing the pressure and the trend ultimately reaches a plateau. The results clearly show that CHF increases with *A*_*r*_, and a heat flux of ~1.8 kW/cm^2^ is reached on structure #5 with the highest *A*_*r*_ (heat flux data as a function of surface superheat temperature are provided in Figure s-3 of Supplementary Materials). Further analysis of the results showed that the ratio of the highest CHF values on structures #4 and #5 versus that of structure #3 (plotted versus ∆P in Fig. 6b) are nearly identical to the ratio of the structures’ respective *A*_*r*_ (cf. Table 2). This was a surprising observation at the onset. Given the substantial difference between the structures dimensions, which often leads to variations in structures thermal efficiency, some difference was expected. However, it was quite fascinating to find out that variations of the wall superheat temperature and hence the heat transfer coefficient (see Table s-2 in the supplementary section) is such that the effective surface area (denoted by ${{\rm{A}}}_{{\rm{r}},{\rm{eff}}}=\frac{{({\rm{S}}+{\rm{W}})}^{2}-{{\rm{W}}}^{2}+{{\rm{W}}}^{2}\times {{\rm{\varepsilon }}}_{{\rm{f}}}}{{({\rm{S}}+{\rm{W}})}^{2}}$, ε_f_ is surface effectiveness), which factors the effect of thermal conductivity and heat transfer coefficient, correlates with the surface maximum CHF value (cf. Table 2). Figure 6b shows that ratio of *A*_*r*, *eff*_ decreases with the heat flux, as it should be, and reaches to ratio of *A*_*r*_ at the limit of CHF. This finding is extremely important as it correlates the highest achievable CHF to the structures thermal conductivity.

**Table 2:** Enhanced area and effective surface area ratio.

Design	${{\bf{q}}}_{{\boldsymbol{CHF}}}^{^{\prime\prime} }$ @ ∆P = 20 kPa (W/cm^2^)	${{\bf{q}}}_{{\boldsymbol{CHF}}}^{^{\prime\prime} }/{{\bf{q}}}_{{\boldsymbol{CHF}}{\boldsymbol{\#}}3}^{^{\prime\prime} }$	*A*_*r*_	*A*_*r*_/*A*_*r#3*_	*A*_*r*, *eff*_ @ ∆P = 20 kPa	*A*_*r*, *eff*_/*A*_*r*_/*A*_*r#3*_
3	~725	1	1.56	1	1.2	1
4	~1025	1.4	2.16	1.4	1.67	1.4
5	~1750	2.4	3.45	2.2	2.56	2.1

The trend discussed here further supports the hypothesis mentioned in the previous section that the local CHF on the surface of these structures is reached as the bubble growth timescale (a function of local wall superheat temperature) becomes shorter than the rewetting timescale. In this case, it appears that a local CHF is reached at the base of structures, which is at the highest superheat temperature, and gradually propagates to the upper levels of the structures with increasing the wall temperature.

## Modeling and Predictions

In a quest for finding the maximum achievable CHF level, a model is developed to accurately predict the CHF values on microstructured surfaces. The comprehensive study presented in the previous sections showed how surface wickability, effective heat transfer area and liquid pressure affect the CHF on microstructured surfaces. The CHF values plotted in Fig. 6, for instance, suggest that in structures with similar surface wickability, increasing the surface area almost linearly enhances the CHF limit, hence it is expected that ${{\rm{q}}}_{{\rm{CHF}}}^{^{\prime\prime} }\,\propto$ surface area. It is also proven that structures with better wickability can reach to higher CHF values (cf. Fig. 5). Additionally, it is recognized that the impact of these parameters directly depends on the liquid pool pressure level. Hence, we hypothesized that CHF on a microstructured surface can be dissected into three parts, as follows:

1 $$\begin{array}{rcl}{{\rm{q}}}_{{\rm{CHF}}}^{^{\prime\prime} }({{\rm{A}}}_{{\rm{r}},{\rm{eff}}},{\rm{\Delta }}{\rm{P}},{\mathscr{W}}) & = & \{{{\rm{q}}}_{{\rm{N}}-{\rm{W}}}^{^{\prime\prime} }({{\rm{A}}}_{{\rm{r}},{\rm{eff}}}=1,{\rm{\Delta }}{\rm{P}} \sim 0,{\mathscr{W}} \sim 0)\\  &  & +{{\rm{q}}}_{{\rm{W}}}^{^{\prime\prime} }({{\rm{A}}}_{{\rm{r}},{\rm{eff}}}=1,{\rm{\Delta }}{\rm{P}},{\mathscr{W}})\}{\times {\rm{F}}}_{{\rm{1}}}({{\rm{A}}}_{{\rm{r}},{\rm{eff}}},{\rm{\Delta }}{\rm{P}})\end{array}$$

where ${\mathscr{W}}$ represents surface wickability. In this equation, ${{\rm{q}}}_{{\rm{N}}-{\rm{W}}}^{^{\prime\prime} }$ represents the maximum amount of heat that can be dissipated from a plain surface (i.e. A_r,eff_ = 1 and ${\mathscr{W}}=0$). Therefore, its value is independent of the surface wickability, liquid pressure and heat transfer area and can only be changed by altering the contact angle. ${{\rm{q}}}_{{\rm{W}}}^{^{\prime\prime} }$, on the other hand, represents heat flux associated with the wicking process and changes with surface wickability and liquid pressure. Finally, F_1_ denotes the effect of enhanced heat transfer area at different liquid pressures. For boiling on a plain surface, ${{\rm{q}}}_{{\rm{W}}}^{^{\prime\prime} }$ and ${{\rm{F}}}_{1}$ are equal to zero and unity, respectively; therefore, ${{\rm{q}}}_{{\rm{CHF}}}^{^{\prime\prime} }={{\rm{q}}}_{{\rm{N}}-{\rm{W}}}^{^{\prime\prime} }$.

### Effect of Surface Wickability

The experimental data presented previously on structures #1–3 are first analyzed to model ${q}_{{\rm{W}}}^{^{\prime\prime} }$. As mentioned earlier, these structures were designed to isolate the effect of surface wickability on CHF. As such, their wickability was varied while their surface area was kept the same. A general formula considering the effects of variable wicking length ($L({\rm{\Delta }}P)$) and non-linear dependence of CHF on wicking velocity (denoted by $\alpha ({\rm{\Delta }}P)$) is formulated as:

2 $${q}_{W}^{^{\prime\prime} }=C\times {(\frac{{{\rm{{\rm K}}}}_{{\rm{wick}}}\times {P}_{c}}{\mu \times L({\rm{\Delta }}P)})}^{\alpha ({\rm{\Delta }}P)}=C\times {(\frac{{{\rm{{\rm K}}}}_{{\rm{wick}}}\times {P}_{c}}{\mu })}^{\alpha ({\rm{\Delta }}P)}\times {(\frac{1}{L({\rm{\Delta }}P)})}^{\alpha ({\rm{\Delta }}P)}$$

**Figure 7:**
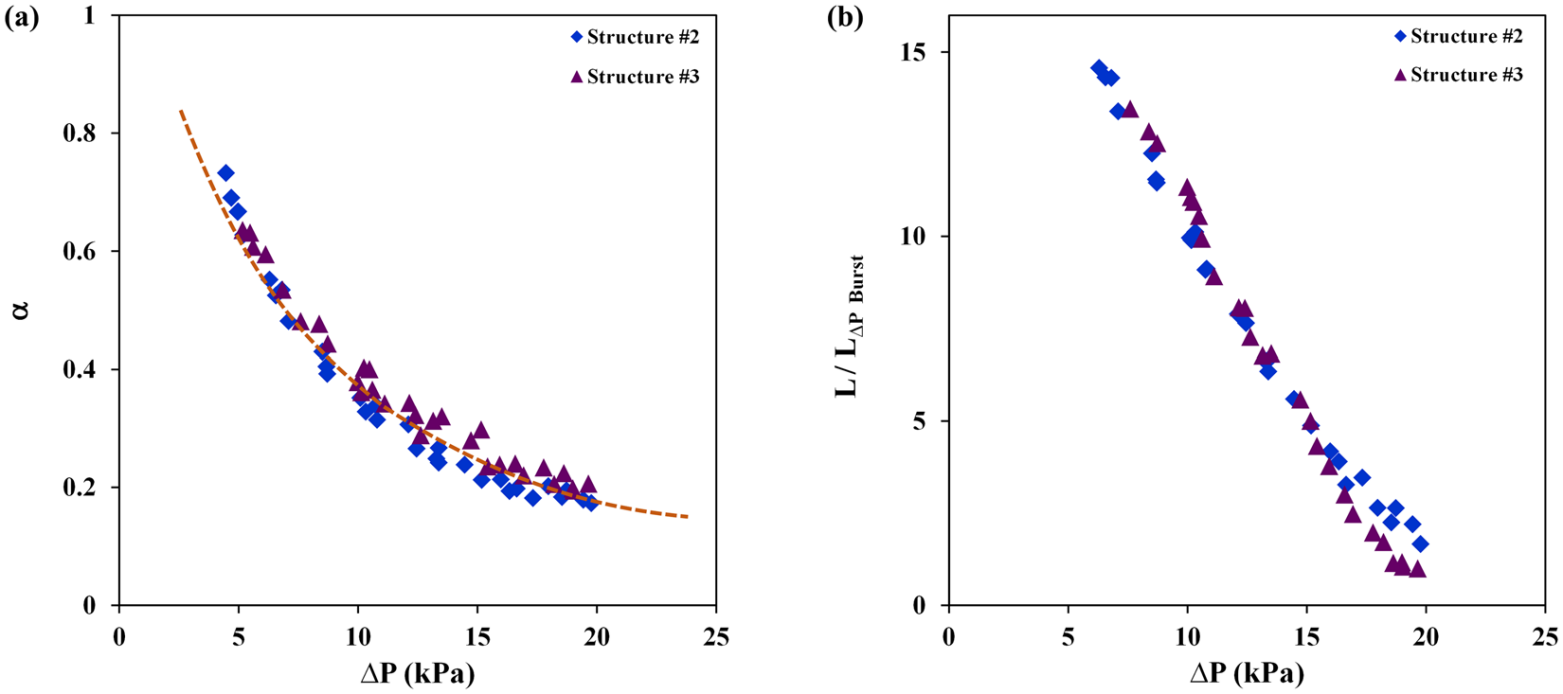
(**a**) CHF dependence on α as a function of liquid pressure (reported values are calculated using equation 3 where *k* = 2, 3 and *j* = 1). (**b**) Normalized wicking length as a function of liquid pressure.

The experimental data are used to calculate *α* and *L*. First, *α* is calculated through cancelling *L* by dividing the CHF values associated with different microstructures at similar applied pressures. The resulting expression for *α* is provided below and the actual values are plotted in Fig. 7a. It should be noted that an *α* equal to one means that CHF is a linear function of surface wickability, as suggested in conventional pool boiling (without the ability to apply pressure) studies^20, 25^. The data suggest that *α* is substantially less than unity at higher pressures where the rewetting process is greatly assisted with pressure.

3 $$\alpha ({\rm{\Delta }}{P}_{i})=[Ln(\frac{{q}_{W,Structure\#k}^{^{\prime\prime} }({\rm{\Delta }}{P}_{i})}{{q}_{W,Structure\#j}^{^{\prime\prime} }({\rm{\Delta }}{P}_{i})})/Ln(\frac{{\{{{\rm{{\rm K}}}}_{{\rm{wick}}}\times {P}_{c}\}}_{Structure\#k}}{{\{{{\rm{{\rm K}}}}_{{\rm{wick}}}\times {P}_{c}\}}_{Structure\#j}})]$$

Equation 2 is then used to estimate the normalized wicking length at different pressures:

4 $$L(\Delta {P}_{i})=L(\Delta P \sim 0)\times {\{\frac{{q}_{W}^{^{\prime\prime} }(\Delta P \sim 0)}{{q}_{W}^{^{\prime\prime} }(\Delta {P}_{i})}\times {(\frac{{{\rm{{\rm K}}}}_{{\rm{wick}}}\times {P}_{c}}{\upsilon })}^{\alpha ({\rm{\Delta }}{P}_{i})-1}\}}^{\frac{1}{\alpha ({\rm{\Delta }}{P}_{i})}}$$

To illustrate the rate of change in wicking length, wicking lengths at different pressures (*L*(*ΔP*)) are normalized by minimum recorded wicking length (*L*(*ΔP*_*Burst*_ ~ 20 *kPa*)) and are plotted in Fig. 7b. The trend shows a linear decline in wicking length with increasing pressure, which clearly confirms the role of pressure on limiting the lateral expansion of bubbles and vapor columns.

5 $$L(\Delta {P}_{i})/L(\Delta {P}_{Burst})\,=[L(\Delta {P}_{i})/L(\Delta P \sim 0)]/[L(\Delta {P}_{Burst})/L(\Delta P \sim 0)]$$

### Effect of Enhanced Heat Transfer Area

The effect of heat transfer area is formulated using the CHF values associated with structures #3–5. As shown earlier, the ratio of the CHF values for these structures starts from unity at low liquid pressure and reaches an asymptotic value that is almost identical to the ratio of effective heat transfer areas of the two structures. Therefore, ${F}_{1}$, which represents the utilized heat transfer area is hypothesized to start from unity at low pressures and reaches a maximum (i.e. ${A}_{r,eff}$) at the highest liquid pressure.

6 $$\{\begin{array}{c}{F}_{1}({A}_{r,eff},\Delta P)\to 1,as\,\Delta P\to 0\\ {F}_{1}({A}_{r,eff},\Delta P)\to {A}_{r,eff},as\,\Delta P\to \Delta {P}_{limit}\end{array}$$

The liquid pressure limit (*ΔP*_*limit*_) introduced in equation 6 represents the conditions at which all heat transfer area is utilized hence *F*_1_ ~ *A*_*r*, *eff*_ and further increase in pressure does not enhance the CHF. *F*_1_ is then formulated as follows (see section S6 in Supplementary Materials):

7 $${F}_{1}=1+({A}_{r,eff}-1)\,f(\Delta P),0 < f(\Delta P) < 1$$

**Figure 8:**
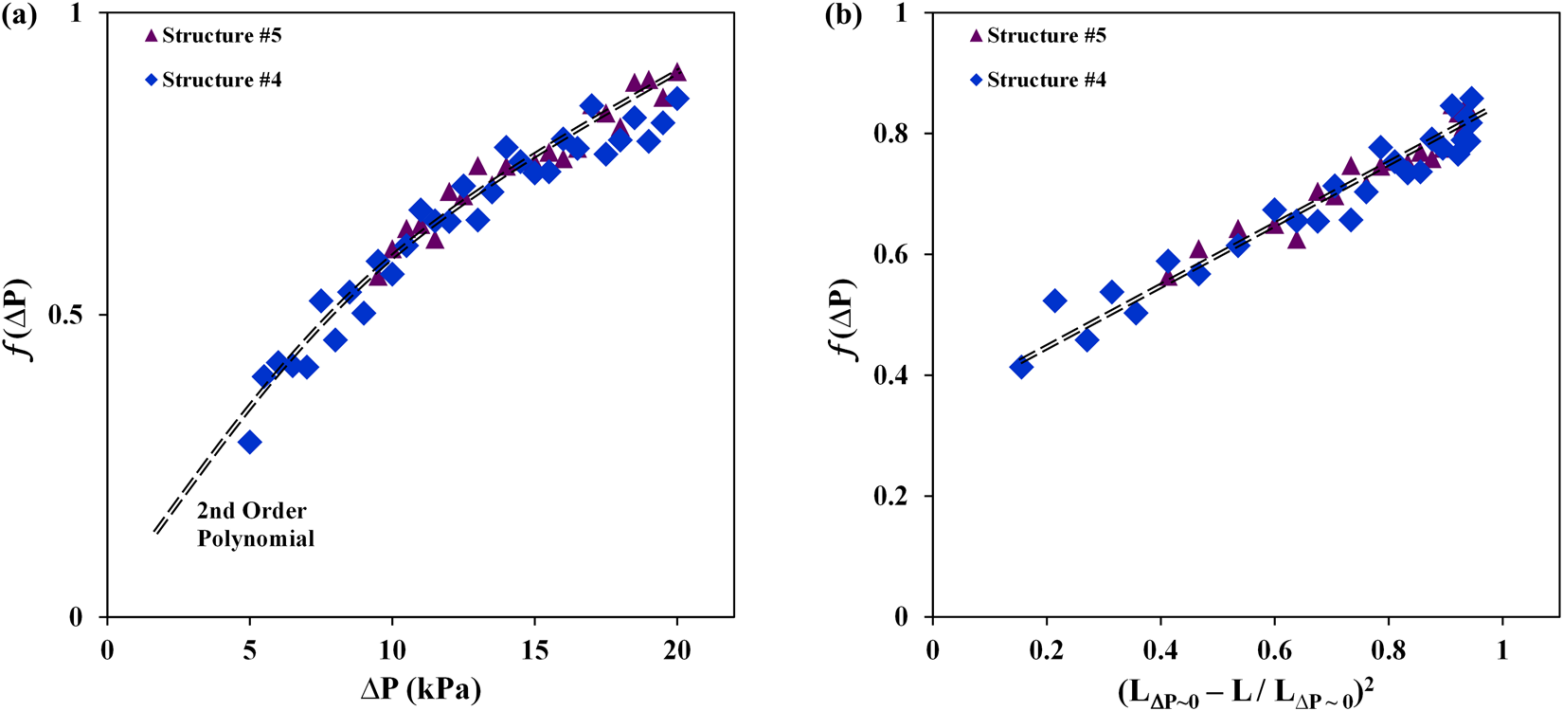
Variations of *f* with (**a**) the applied liquid pressure and (**b**) available wicking area (values for *f*(Δ*P*) are calculated by normalizing the CHF values associated with structures 4 & 5 with that of structure #3).

Using the experimental data, presented in Fig. 6, *f*(Δ*P*) = (*F*_1_ − 1)/(*A*_*r*, *eff*_ − 1) was calculated and plotted as a function of liquid pressure (${\rm{\Delta }}P$) in Fig. 8a. This parameter (*f* ) represents the ratio of actual surface area used for removing heat (*F*_1_) to the effective heat transfer area (*A*_*r*, eff_) achievable at the same pressure, which enhances with increasing the liquid pressure and is expected to reach to unity at higher pressures. Interestingly, the calculated values are almost identical for different structures and only change with Δ*P* which verifies the independency of this parameters from the structure geometry. A second order polynomial in the form of $a\Delta P+b\Delta {P}^{2}$ was used to estimate this function with less than 10% relative error.

The changes in *f*(Δ*P*) as a function of ∆P can be further explained by considering the role of applied liquid pressure on reducing the effective wicking length and potentially providing more surface area. As shown previously in Figs 1b and 7b, increasing the liquid pressure limits the lateral expansion of bubbles and reduces the wicking length which can potentially result in enhancing the available surface area for heat transfer. It is hypothesized that the available surface area should be related to the area wetted due to increased liquid pressure. Figure 8b plots *f*(Δ*P*) as a function of ${({L}_{\Delta P \sim 0}-L/{L}_{\Delta P \sim 0})}^{2}$, which represents the wetted surface area, and shows a linear relationship between the two parameters. This behavior proves, again, the role of applied liquid pressure on increasing surface area and reducing the wicking length.

Finally, the value of ${q}_{N-W}^{^{\prime\prime} }$ is estimated to be ~98 *W*/*cm* ^2^ based on the best fit to all experimental data. This value is in a reasonable agreement with the experimental data available in the literature for pool boiling CHF on plain surfaces^54–56^.

**Figure 9:**
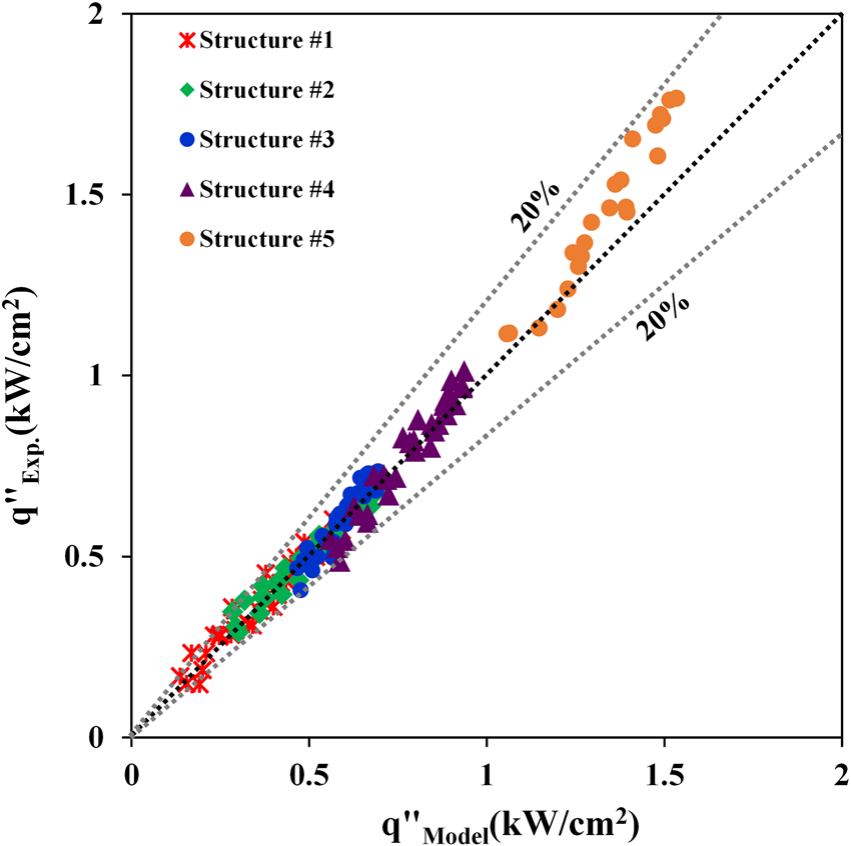
Comparison of experimental and modeled CHF values (the maximum relative error is less than 20%).

As discussed, different elements of the model were designed based on a detailed analysis of various experimental data. Figure 9 presents an overall comparison between the model predictions and all experimental data. As can be seen, the model predicts the CHF performance of different surfaces with less than 20% relative error.

## Closure

The results presented in previous sections suggest that to further enhance CHF, the effective surface area must be increased. One approach for reaching this goal involves reducing the spacing between the pillars (i.e. increasing the pillars density). However, decreasing the spacing reduces the structure wickability, and the model suggests that wickability becomes the limiting factor in this regime. To counter this effect, the pillars can be made thin and tall but this approach reduces the fin efficiency (*η*_*f*_) and effectiveness (*ε*_*f*_) which in turn decreases the effective surface area. To solve this problem and keep fin efficiency and effectiveness within acceptable margins while employing tall and thin pillars, it is essential to increase the thermal conductivity of the base material.

**Figure 10:**
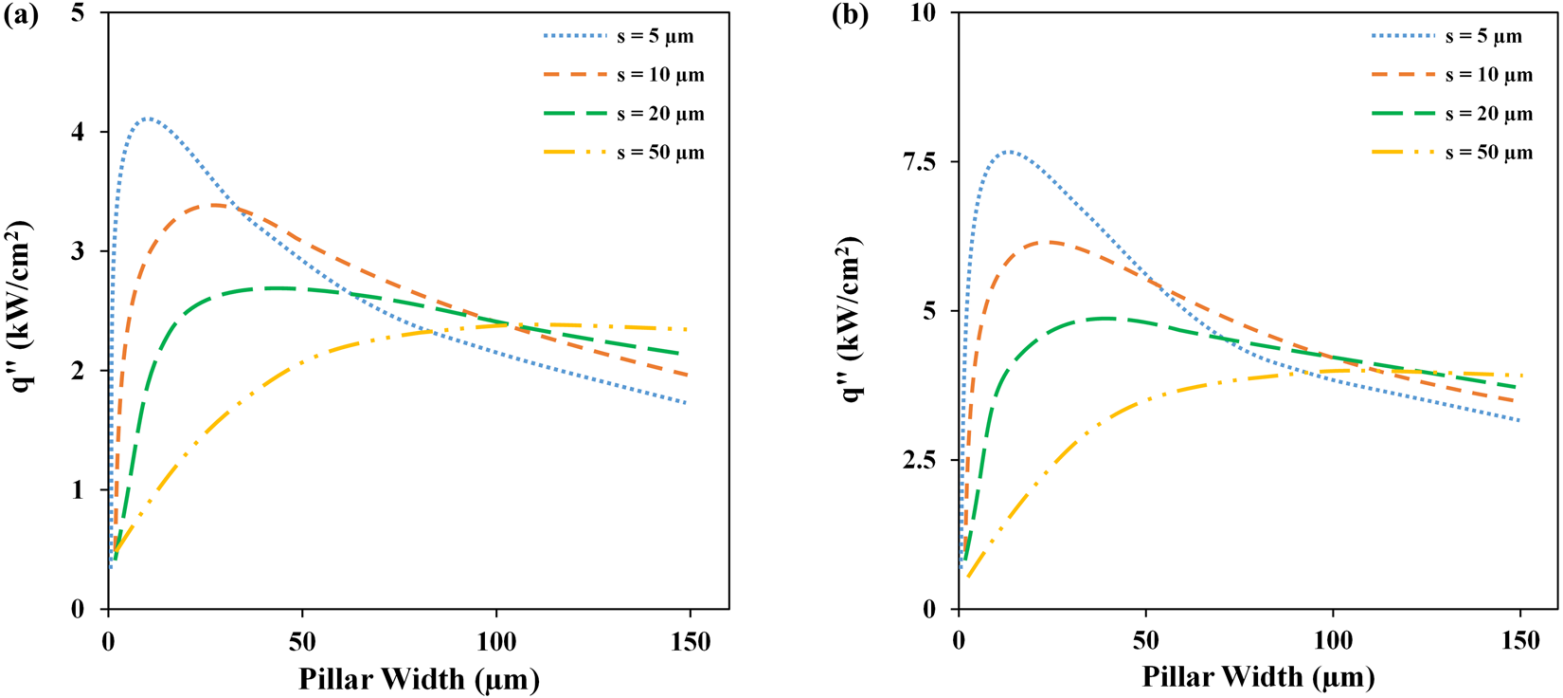
CHF values estimated from the model for a surface with (**a**) copper and (**b**) diamond structures.

Considering these design guidelines, the model developed in the previous section was then used in a parametric study to determine the maximum achievable CHF from structured surfaces made from copper and diamond. In this study, the upper limit of pressure is fixed at 20 kPa (which is equal to the burst pressure of our best membrane). The parametric study reveals an interesting interplay between different parameters affecting the structure CHF performance. The results clearly show that depending on the pillars dimensions and spacing, CHF can become limited by wickability, heat transfer area or the thermal conductivity of the surface structures material. The outcome of the parametric study is summarized in Fig. 10. It should be noted that a heat transfer coefficient of 200 kW/m^2^ K is used in estimating the pillars thermal efficiency and effectiveness. The results suggest that a copper structure with 10-µm-wide and 150-µm-tall pillars spaced 5 µm apart may reach a CHF of ~4 kW/cm^2^ (Fig. 10a) and a diamond structure with 15-µm-wide and 250-µm-tall pillars spaced 5 µm apart may reach a CHF of 7–8 kW/cm^2^ (Fig. 10b).

## Conclusion

A new approach was implemented to manipulate dynamics of liquid and vapor phases interactions in proximity of the heated surface. The approach allowed controlling rewetting and delaying CHF on different structured surfaces. This new degree of freedom in conjunction with a careful design of a set of surfaces with different wickability and heat transfer area enabled a better understanding of the CHF physics. The parameters influencing CHF were clearly identified and their relative impact was precisely quantified. It was demonstrated that CHF can be varied by tailoring the surface wickability, heat transfer area, and applied pressure, and each of these factors can limit the CHF depending on the surface structure design and heat flux level. It was shown that surface natural ability to rewet itself (i.e. wickability) plays a key role at low heat flux. However, to reach high CHF levels, pressure-assisted re-wetting is required to overcome strong momentum forces generated at the bubbles-liquid interface. It was revealed that increasing the surface area can enhance CHF only when surface is sufficiently rewetted. This behavior was explained by showing the effect of liquid pressure on wickability length, where the wicking length was decreased by increasing the liquid pressure. It was argued that the thermal conductivity of the base material ultimately limits the CHF, as thermal effectiveness of surface structures decline, and diamond surface structure may enable a CHF of 7–8 kW/cm^2^. Considering that the effect of different surface properties on CHF are now explained, the results of this study may inspire alternative approaches to control bubble-liquid interfacial dynamics to enhance CHF.

## Electronic supplementary material

Supplementary Information
