# Supplementary material for: A New Paradigm for Understanding and Enhancing the Critical Heat Flux (CHF) Limit
Source: Sci Rep. 2017 Jul 12;7:5184. doi: 10.1038/s41598-017-05036-2 (PMC5507906; doi:10.1038/s41598-017-05036-2)
Supplement: Supplementary file 1 — Supplementary Information [file 41598_2017_5036_MOESM1_ESM.pdf]

SUPPLEMENTARY MATERIALS

A New Paradigm for Understanding and Enhancing the Critical Heat Flux (CHF) Limit

Abdolreza Fazeli and Saeed Moghaddam*

Department of Mechanical and Aerospace Engineering, University of Florida, Gainesville, FL 32611, USA

*Email: saeedmog@ufl.edu

S1. Experimental setup

Fig. s-1 depicts a schematic of the test loop used in this study. Water is used as the test fluid and is supplied to the heat sink using a micropump (Model MP6, manufactured by Bartels Mikro-technik GmbH). This micropump can independently control the supplied liquid pressure and flow rate. Inside the test device (schematically shown in Fig. s-2), liquid is delivered to the heated surface via a 1 × 1 mm^2 cross-section channel. The generated vapor exiting the device is condensed on the internal surface of the chamber cooled by a thermoelectric cooler installed on the chamber external surface. A more detailed description of the experimental setup is presented in our previous work[1].

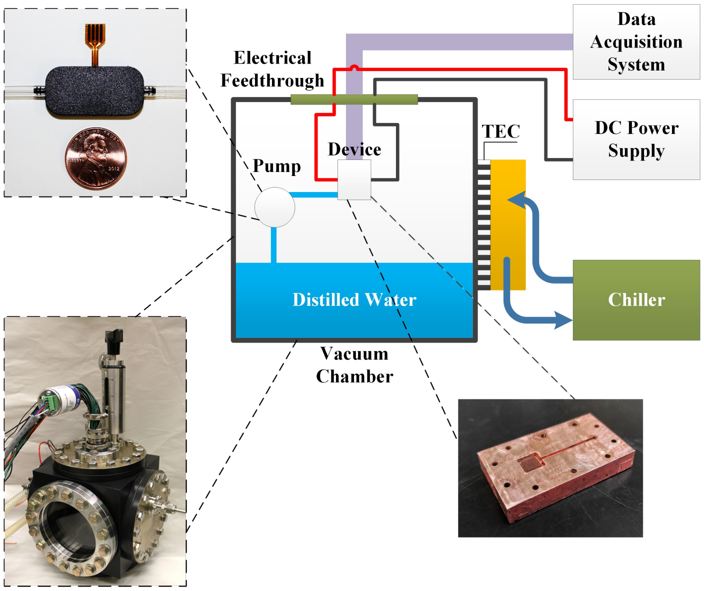

Figure s-1: Schematic of the test loop.

To ensure that boiling occurs only on the structured surface, the side walls of the liquid delivery channel were covered with a 200-μm-thick layer of epoxy, since a low thermal conductivity surface is shown to inhibit boiling[2]. The liquid supply line was initially machined with a 1.2 × 1.4 mm^2 cross-section. The

channel was then filled with epoxy and cured for 6 hours. The supply channel was re-machined with a cross-section of 1.0 × 1.0 mm^2 within the epoxy, leaving a 200-µm-thick layer of epoxy covering all channel walls.

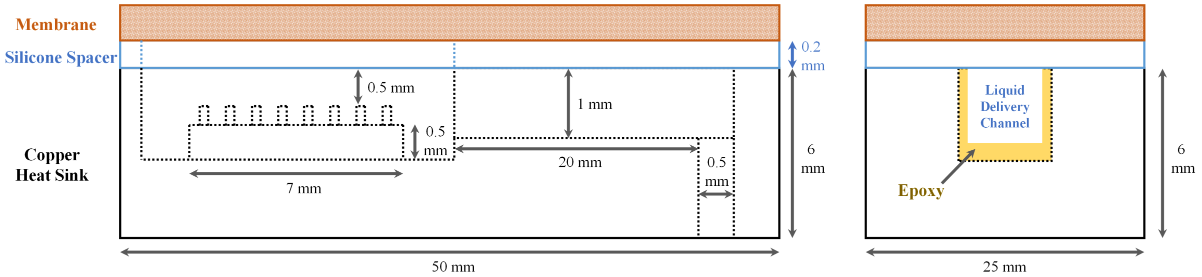

Figure s-2: Side view of the assembled copper heat sink with silicone spacer and membrane. Drawings are not to scale.

The copper block used in this study is shown in Fig. s-3. The copper heat sink is brazed to a heating block which provides the required heat input and measures the temperature of the heated surface. The generation and accurate measurement of heat fluxes at the 1-5 kW/cm^2 level is a nontrivial task. In this study, we utilized copper blocks with imbedded cartridge heaters to produce the required heat flux. In the following, details of the heating and measurement platform used for the preliminary studies are described.

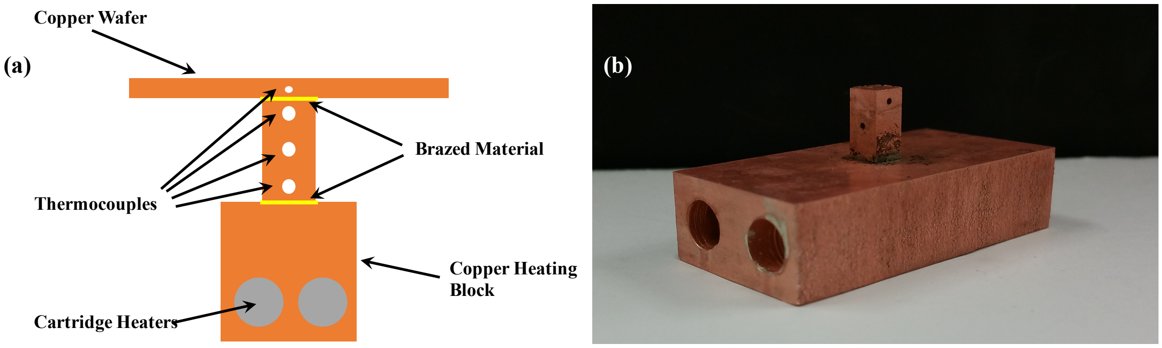

Figure s-3: Heater block and device assembly; (a) A schematic showing device assembly on the heater block and (b) heater block used in preliminary experiments.

The heating block consists of two 500 W cartridge heaters embedded in a copper block (cf. Fig. s-3). In this design, the heater block is attached to the test chip with a copper column. Three thermocouples are installed within the column to measure the temperature and calculate heat flux using the following equation.

$$q" = -k\frac{dT}{dx} \sim k\times(3T_1 - 4T_2 + T_3)/2\Delta x \qquad (s\text{-}1)$$

For testing at extremely high heat flux conditions, dimensions of the heater block must be optimized to maintain its maximum temperature below the melting point of the brazing materials which is 800°C to 900°C for majority of copper alloys [3]. Consequently, the heater block (cf. Fig. s-3 b) was designed to keep the maximum temperature below the melting point. A numerical simulation conducted to estimate the maximum temperature of the heater block showed that the maximum temperature could reach to ~750°C at a heat flux of 2 kW/cm^2 over a 7×7 mm^2 heat transfer area (cf. Fig s-4).

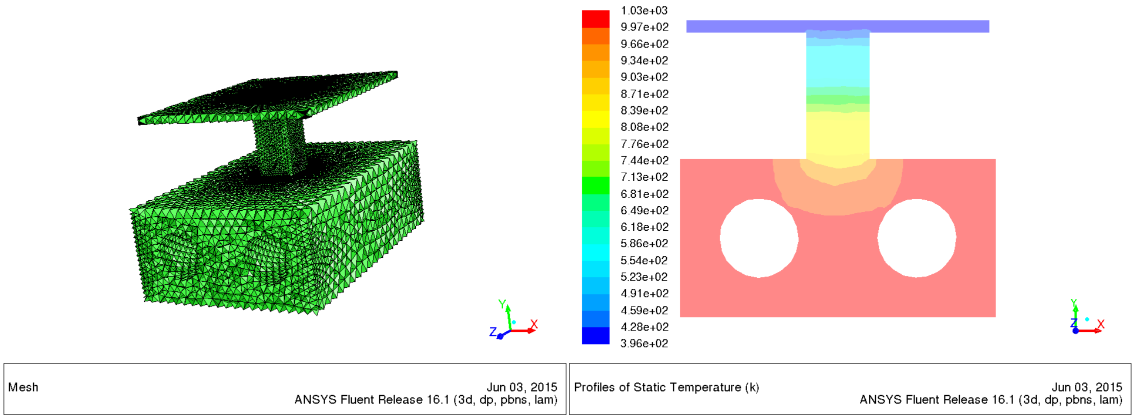

Figure s-4: Numerical modeling of temperature distribution with the heater block; (a) Simulation domain and (b) Temperature contours (temperature values are in Kelvin).

S2. Calculation of capillary pressure and permeability

There are numerous models available in the literature which can be used to estimate capillary pressure (ΔP) and and structure permeability ($\mathcal{K}_v$) [4–8]. Ravi et al. [9] compared these models and showed that the best model for measuring liquid flow rate within the wicking arrays, and consequently liquid front velocity, can be achieved by combining the permeability model by Byon and Kim [7] and the capillary model by Xiao et al. [8] with Darcy's law.

The permeability model developed by Byon and Kim [7] was based on an earlier two-dimensional permeability model proposed by Sangani and Acrivos [6]. They modified this two-dimensional model to include the 3-D effects associated with the height of pillars and liquid meniscus curvature on the permeability of a microstructured surface:

$$\mathcal{K}_v = \left(\frac{h_{eff} + \dfrac{\phi d}{4(1-\phi)}}{h + \dfrac{\phi d}{4(1-\phi)}}\right)\left(\frac{h_{eff}}{h}\right)\left[1 - \frac{exp\left(2\sqrt{\dfrac{\phi}{K_{2D}}}h_{eff}\right) - 1}{\sqrt{\dfrac{\phi}{K_{2D}}}h_{eff}exp\left(2\sqrt{\dfrac{\phi}{K_{2D}}}h_{eff}\right) - 1}\right]K_{2D} \qquad (s\text{-}2)$$

where

$$h_{eff} = h - d\times \left\{ \begin{array}{c} 0.01476 + 0.85009cos\theta + 0.215\frac{d}{p} + 0.18979\,cos^2\,\theta - 3.46929\frac{d}{p}cos\theta \\ -0.28868\left(\frac{d}{p}\right)^2 + 1.05357\frac{d}{p}cos^2\,\theta + 3.12583\left(\frac{d}{p}\right)^2 cos\theta - 1.4243\left(\frac{d}{p}cos\theta\right)^2 \end{array} \right\} \quad (s\text{-}3)$$

Capillary pressure in the microstructured surfaces was estimated using a semi-analytical model developed by Xiao et al. [8]. In this model, first a surface energy minimization algorithm [10] was utilized to determine the shape and energy of a free meniscus. Then, capillary pressure was calculated as the change in surface energy per unit volume. The simulation results were experimentally verified by determining the shape of the meniscus formed by water in unit cells of similar dimensions.

$$P_c = \frac{\Delta E}{\Delta V} = \frac{(\sigma rf cos\theta)\pi dh + \sigma cos\theta\left(p^2 - \frac{\pi d^2}{d}\right) - \sigma A_m}{\Delta V} \quad (s\text{-}4)$$

$$A_m = \left(s^2 - \frac{\pi d^2}{4}\right)\left\{ \begin{array}{c} 0.43 + 0.73rfcos\theta + 3.76\left(\frac{d}{s}\right) - 0.046(rfcos\theta)^2 \\ -5.53(rfcos\theta)\left(\frac{d}{s}\right) - 4.05\left(\frac{d}{s}\right)^2 - 0.124(rfcos\theta)^3 \\ +1.77(rfcos\theta)^2\left(\frac{d}{s}\right) + 4.66(rfcos\theta)\left(\frac{d}{s}\right)^2 \end{array} \right\} \quad (s\text{-}5)$$

$$\Delta V = h\left(s^2 - \frac{\pi d^2}{4}\right) - w\left(s^2 - \frac{\pi d^2}{4}\right)\left\{ \begin{array}{c} -0.175 - 0.345rfcos\theta + 4.07\left(\frac{d}{s}\right) + 0.924(rfcos\theta)^2 \\ -5.83(rfcos\theta)\left(\frac{d}{s}\right) - 2.80\left(\frac{d}{s}\right)^2 - 0.439(rfcos\theta)^3 \\ +2.41(rfcos\theta)^2\left(\frac{d}{s}\right) + 2.71(rfcos\theta)\left(\frac{d}{s}\right)^2 \end{array} \right\} \quad (s\text{-}6)$$

In these equations, A_m represents the area of the meniscus, determined using the Surface Evolver algorithm [10], and rf accounts for the surface roughness of the pillar wall. These models consider the effect of three-dimensional menisci on permeability and capillary pressure and can estimate the wickability of the microstructure with higher accuracy compared to other models [4–6]. The average difference between wicking values computed using the overall model and experimental data was about 18%.

S3. Effect of membrane permeability on CHF

In order to study the effect of membrane permeability on critical heat flux, we utilized three membranes with an order of magnitude change in permeability. Table s-1 lists the permeability of these membranes. Membrane permeability values are measured experimentally using Gurley method by the manufacturers and were reported in the membranes datasheets.

Table s-1: Membrane properties

Membrane	Material	Permeability ($l/hr/cm^2/kPa$)
#1	Polyethersulfone (PES)	0.2
#2	Polytetrafluoroethylene (PTFE)	0.7
#3	Acrylic Copolymer(Versapor)	1.1

It was shown in the manuscript that in the regions where membrane permeability limits the performance of microstructured surface (i.e. highlighted area in Fig. 3b) the ratio of critical heat flux to liquid pressure is relatively constant. This behavior can be elucidated by examining the relation between membrane mass transfer limit and critical heat flux:

$$\left.\begin{aligned} \dot{m}''_{mem} &= K_{mem}\times\Delta P \\ q''_{CHF} &= \dot{m}''_{mem}\times h_{fg} \end{aligned}\right\} \rightarrow {q''_{CHF}}/{\Delta P} \propto K_{mem} \qquad (s\text{-}7)$$

The ratio of the experimental values measured for ${q''_{CHF}}/{\Delta P}$ almost matches the ratio of membrane permeability of each membrane, which further proves our discussion.

S4. Maximum reported CHF

Figure s-3 provides heat flux versus superheat data for structure #5 at applied liquid pressure of 20.3 kPa. The heat transfer area ratio for the structure was the maximum (A_r = 3.54), which corresponded to a maximum critical heat flux value of ~1760 W/cm^2 at wall superheat less than 36 ℃. The CHF value reached by this structure is an order of magnitude higher than values reported in the literature and clearly shows the importance of applied liquid pressure and heat transfer area on pushing the CHF limit.

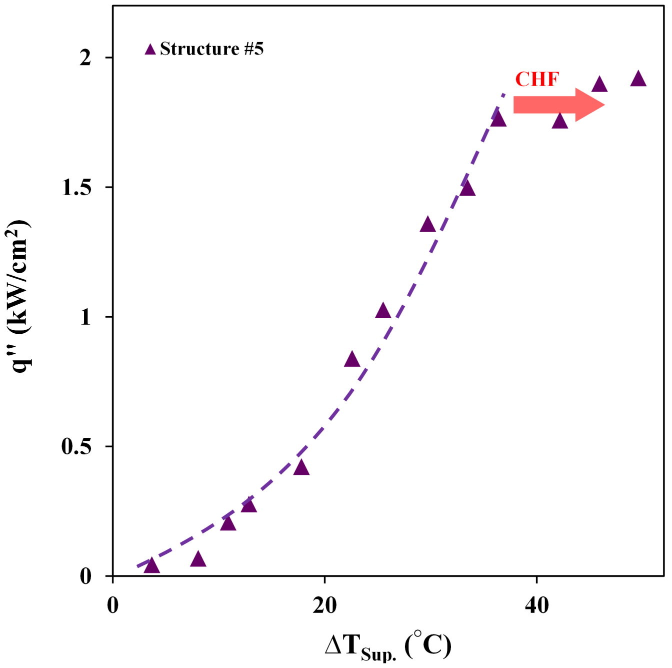

Figure s-5: Boiling curve for structure #5 as a function of wall superheat

S5. Calculation of effective surface area ratio

The enhanced area ratio A_r introduced in the "Experimental Studies" section calculates the overall surface area of the microstructured surfaces; however, it does not reflect the effects of heat transfer on the effective surface area and therefore is not an accurate representation of the available heat transfer area. In order to consider the effects of surface geometry, heat transfer coefficient and also material thermal conductivity on added surface area, we introduced effective surface area ratio, defined as:

$$A_{r,eff} = \frac{(S+W)^2 - W^2 + W^2 \times \varepsilon_f}{(S+W)^2} \qquad \textit{(s-8)}$$

where ε_f denotes the effectiveness of microstructures and is calculated using the following equation:

$$\varepsilon_f = \sqrt{\frac{kP}{hA_c}} \times \frac{\sinh(mL) + \left(h/_{mk}\right)\cosh(mL)}{\cosh(mL) + \left(h/_{mk}\right)\sinh(mL)} \qquad \textit{(s-9)}$$

in this equation h denotes heat transfer coefficient and should be calculated separately. For each test, h was calculated using the following equation solved by an iterative-Matlab code:

$$q''_{CHF} = h\left(1 + \eta_f \times A_r\right) \times \Delta T_{sup} \qquad (s\text{-}10)$$

where

$$\eta_f = \sqrt{\frac{kP}{hA_c}} \times \frac{\sinh(mL) + \left(h/_{mk}\right)\cosh(mL)}{\cosh(mL) + \left(h/_{mk}\right)\sinh(mL)} \qquad (s\text{-}11)$$

Table s-2 lists the values for heat transfer coefficient, efficiency, effectiveness and effective surface area ratio of structures #3-5 at ΔP ~ 20kPa. It can be seen that at this pressure, the ratio of effective surface areas ($^{A_{r,eff,\#j}}/_{A_{r,eff,\#i}}$) is almost identical to the ratio of enhanced area ($^{A_{r,\#j}}/_{A_{r,\#i}}$) calculated previously. This result proves the applicability of $A_{r,eff}$ for determining the effective surface area of each microstructure and also shows the significance of material thermal conductivity and geometrical dimensions on increasing the effective surface area.

Table s-2: Effective surface area ratio of structures #3-5 at ΔP ~ 20 kPa

Design	HTC ($kW/m^2.K$)	ΔT_{CHF}(°C)	Efficiency (η_f)	Effectiveness (ε_f)	$A_{r,eff}$	A_r
3	~270	20.4	0.39	10.6	1.2	1.56
4	~310	26.1	0.65	3.31	1.67	2.16
5	~120	35.9	0.64	9.16	2.56	3.45

S6. Effect of heat transfer area on CHF

As shown in the manuscript (cf. Eq. (1)), in order to consider the effects of enhanced heat transfer area on increasing CHF, q''_{CHF} can be reformulated as follows:

$$q''_{CHF} = F_1(A_r, \Delta P) \times \left\{q''_{N-W} + q''_W\right\} \qquad (s\text{-}12)$$

$$\begin{cases} F_1(A_r, \Delta P) \to 1\,, & as\ \Delta P \to 0 \\ F_1(A_r, \Delta P) \to A_{r,eff}\,, & as\ \Delta P \to \Delta P_{limit} \end{cases} \qquad (s\text{-}13)$$

Where F_1 denotes the surface area utilized for heat transfer at different liquid pressure. This equation suggests that at extreme conditions ($\Delta P \to 0$ or $\Delta P \to \Delta P_{limit}$), the heat transfer area for different microstructures is only a function of surface geometries and are independent of liquid pressure (i.e. it

starts off from unity at extremely low liquid pressure and reaches a limiting value ($A_{r,eff}$) at elevated pressures). It is therefore hypothesized that F_1 can be normalized with respect to these limiting values to reach an inclusive form (f) that is independent of geometrical dimensions of microstructures and can be estimated as a function of liquid pressure:

$$\frac{F_1(s,d,h,\Delta P)-1}{A_{r,eff}(s,d,h)-1} = f(\Delta P) \qquad \textit{(s-14)}$$

To check the validity of this hypothesis, we used equation (1) to compare the thermal performance of tested devices with similar wickability:

$$\frac{q''_{CHF,\#j}}{q''_{CHF,\#i}} = F_{1,\#j}\,(s,d,h,\Delta P) \Big/ F_{1,\#i}\,(s,d,h,\Delta P) \qquad \textit{(s-15)}$$

Which resulted in

$$f(\Delta P) = \left[\left(1 - \frac{q''_{CHF,\#j}}{q''_{CHF,\#i}}\right) \Big/ \left((A_{r,eff,\#j} - 1) \times \frac{q''_{CHF,\#j}}{q''_{CHF,\#i}} - (A_{r,eff,\#i} - 1) \right) \right] \qquad \textit{(s-16)}$$

Fig. s-6 shows f(ΔP) as a function of applied liquid pressure for micro-structures #2 and #3 compared with micro-structure #1. As predicted, f(ΔP) is only a function of applied liquid pressure and microstructural geometries don't have any effect on it, which further proves our hypothesis. A second-order polynomial is then used to predict (f) as a function of applied liquid pressure (ΔP) and coefficients a and b are calculated to be:

$$f(\Delta P) = a\Delta P + b\Delta P^2 \qquad \textit{(s-17)}$$

where $\mathrm{a} = 0.076\ (\mathrm{kPa}^{-1}), \mathrm{b} = -0.0017\ (\mathrm{kPa}^{-2})$

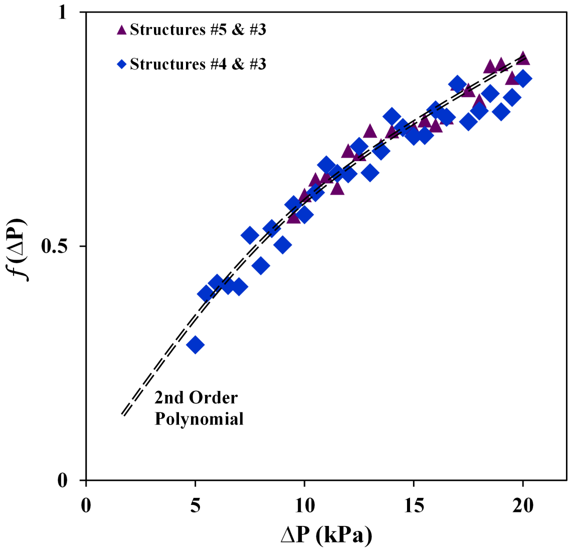

Figure s-6: f(ΔP), plotted by using data from devices 1-3 as a function of liquid pressure (independent of surface geometry)

S7. Uncertainty Analysis

Temperatures in the heating block and copper heat sink are recorded using T-type thermocouples with an uncertainty of ±0.5 °C. A gauge pressure transducer (Omega PX26) is utilized to measure the pressure difference across the membrane with ±0.50% (full scale) uncertainty. The uncertainties associated with other instruments are listed in Table s-1 (obtained from the manufacturer's datasheet).

Table s-1: Variables uncertainties

Variable	Uncertainty
Voltage	±0.05%
Current	±0.20%
Heated area	±0.20%

The uncertainty associated with heat dissipated through phase change process (q'') is due to uncertainty in the thermal conductivity and spacing measurements as well as temperature readings. Equation (s-18) provides the heat flux uncertainty.

$$\frac{\delta q}{q} = \sqrt{\left(\frac{\delta k}{k}\right)^2 + \left(\frac{\delta \Delta T}{\Delta T}\right)^2 + \left(\frac{\delta \Delta x}{\Delta x}\right)^2} \quad \text{(s-18)}$$

where $\Delta T = 3T_1\text{-}4T_2 + T_3$ and $\delta\Delta T = \sqrt{(3\delta T)^2 + (4\delta T)^2 + (\delta T)^2} \sim 4.12\delta T$. The heat flux uncertainty is found to be ±49.8% at the lowest reported CHF (~190 W/cm^2). The uncertainty then drops to ±7.9% and ±5.4% at CHF ~ 1200 W/cm^2 and 1766 W/cm^2, respectively. The uncertainty in measuring heat flux, area ratio and superheat temperature then dictates the uncertainty of the estimate heat transfer coefficient (HTC). Equations (s-2) – (s-4) can be used to calculate uncertainty associated with HTC.

$$X(q,h,A_r,\Delta T_{sup.}) = h\times(1 + \eta_f A_r)\Delta T_{sup} - q = 0 \tag{s-19}$$

$$\begin{cases} \dfrac{\partial X(q,h,A_r,\Delta T_{sup.})}{\partial q} = \dfrac{\partial h}{\partial q}\times(1 + \eta_f A_r)\Delta T_{sup.} + h\times\left(\dfrac{\partial \eta_f}{\partial h}\dfrac{\partial h}{\partial q}A_r\right)\Delta T_{sup.}\text{-}1 = 0 \\ \dfrac{\partial X(q,h,A_r,\Delta T_{sup.})}{\partial A_r} = \dfrac{\partial h}{\partial A_r}\times(1 + \eta_f A_r)\Delta T_{sup.} + h\times\left(\dfrac{\partial \eta_f}{\partial h}\dfrac{\partial h}{\partial A_r}A_r\right)\Delta T_{sup.} + h\times\eta_f\Delta T_{sup.} = 0 \\ \dfrac{\partial X(q,h,A_r,\Delta T_{sup.})}{\partial \Delta T_{sup}} = \dfrac{\partial h}{\partial \Delta T_{sup.}}\times(1 + \eta_f A_r)\Delta T_{sup.} + h\times\left(\dfrac{\partial \eta_f}{\partial h}\dfrac{\partial h}{\partial \Delta T_{sup.}}A_r\right)\Delta T_{sup.} + h\times(1 + \eta_f A_r) = 0 \end{cases} \tag{s-20}$$

$$\delta h^2 = \left(\frac{\partial h}{\partial q}\times\delta q\right)^2 + \left(\frac{\partial h}{\partial A_r}\times\delta A_r\right)^2 + \left(\frac{\partial h}{\partial \Delta T_{sup.}}\times\delta\Delta T_{sup.}\right)^2 \tag{s-21}$$

The HTC uncertainty is found to be ±52.4% at lowest reported CHF ~ 190 W/cm^2. The uncertainty then drops to ±9.3% and ±7.4% at CHF ~ 1200 W/cm^2 and 1766 W/cm^2, respectively. Similarly, the uncertainty associated with the effective surface area can be calculated using the following equation.

$$\delta A_{r,eff}^2 = \left(\frac{\partial A_{r,eff}}{\partial S}\times\delta S\right)^2 + \left(\frac{\partial A_{r,eff}}{\partial W}\times\delta W\right)^2 + \left(\frac{\partial A_{r,eff}}{\partial \varepsilon_f}\times\delta\varepsilon_f\right)^2 \tag{s-22}$$

Our calculation showed that maximum uncertainty of effective surface area for different microstructures reduces from ±56.4% at CHF ~ 190 W/cm^2 to ±11.8% and ±9.7% at CHF ~ 1200 W/cm^2 and 1766 W/cm^2, respectively. Each test was conducted for at least four times to ensure the repeatability of the presented data. The heat flux values reported is the average of data points recorded at each test point.

References

1. Fazeli, A., Mortazavi, M. & Moghaddam, S. Hierarchical biphilic micro/nanostructures for a new generation phase-change heat sink. *Appl. Therm. Eng.* **78,** 380–386 (2015).
2. Rahman, M. M., Pollack, J. & McCarthy, M. Increasing Boiling Heat Transfer using Low Conductivity Materials. *Sci. Rep.* **5,** 13145 (2015).
3. Havis, M. T. Brazing copper and copper alloys. *PRACTICAL WELDING TODAY* (2007).
4. Tamayol, A. & Bahrami, M. Analytical determination of viscous permeability of fibrous porous media. *Int. J. Heat Mass Transf.* **52,** 2407–2414 (2009).
5. Tamayol, A. & Bahrami, M. Transverse permeability of fibrous porous media. *Phys. Rev. E*

83, 46314 (2011).
6. Sangani, A. S. & Acrivos, A. Slow flow past periodic arrays of cylinders with application to heat transfer. *Int. J. Multiph. Flow* **8,** 193–206 (1982).
7. Byon, C. & Kim, S. J. The effect of meniscus on the permeability of micro-post arrays. *J. Micromechanics Microengineering* **21,** 115011 (2011).
8. Xiao, R., Enright, R. & Wang, E. N. Prediction and Optimization of Liquid Propagation in Micropillar Arrays. *Langmuir* **26,** 15070–15075 (2010).
9. Ravi, S., Horner, D. & Moghaddam, S. Monoporous micropillar wick structures, I-Mass transport characteristics. *Appl. Therm. Eng.* **73,** 1371–1377 (2014).
10. Brakke, K. A. The Surface Evolver. *Exp. Math.* **1,** 141–165 (1992).
